# Duration estimates within a modality are integrated sub-optimally

**DOI:** 10.3389/fpsyg.2015.01041

**Published:** 2015-08-12

**Authors:** Ming Bo Cai, David M. Eagleman

**Affiliations:** Laboratory for Perception and Action, Department of Neuroscience, Baylor College of MedicineHouston, TX, USA

**Keywords:** duration perception, cue integration, memory decay, Bayesian inference, temporal frequency, time order error, just noticeable difference

## Abstract

Perceived duration can be influenced by various properties of sensory stimuli. For example, visual stimuli of higher temporal frequency are perceived to last longer than those of lower temporal frequency. How does the brain form a representation of duration when each of two simultaneously presented stimuli influences perceived duration in different way? To answer this question, we investigated the perceived duration of a pair of dynamic visual stimuli of different temporal frequencies in comparison to that of a single visual stimulus of either low or high temporal frequency. We found that the duration representation of simultaneously occurring visual stimuli is best described by weighting the estimates of duration based on each individual stimulus. However, the weighting performance deviates from the prediction of statistically optimal integration. In addition, we provided a Bayesian account to explain a difference in the apparent sensitivity of the psychometric curves introduced by the order in which the two stimuli are displayed in a two-alternative forced-choice task.

## Introduction

Estimating how long an event lasts is a perceptual capacity that we utilize in daily life. For example, we distinguish words with similar sounds, such as “sheep” and “ship,” based on the duration of a syllable; a salesman can infer a customer's interest by how long the customer gazes on each item; we judge internet speed based on the time it takes to load a webpage; various electric devices signal different messages to us by the duration of a beep or flash. However, the mechanisms by which the brain estimates a duration is still unclear (For an non-exhaustive list of recent reviews on duration perception, see Eagleman, 2008; Ivry and Schlerf, 2008; Grondin, 2010; Merchant et al., 2013). A traditional view of duration perception is that the brain possesses a dedicated “internal clock” (Treisman, 1963; Gibbon, 1977). In this view, duration perception is less dependent on low-level sensory processing. However, recent psychophysical studies have revealed that perceived duration can, in fact, be influenced by various properties of a visual stimulus, such as temporal frequency or speed of motion (Brown, 1995; Kanai et al., 2006; Kaneko and Murakami, 2009; Tomassini et al., 2011; Kline and Reed, 2013), change of speed (Carrozzo and Lacquaniti, 2012), numerosity (Long and Beaton, 1981; Xuan et al., 2007), contrast (Long and Beaton, 1980; Xuan et al., 2007), spatial frequency (Aaen-Stockdale et al., 2011), and looming (van Wassenhove et al., 2008). The fact that duration perception is influenced by so many low-level sensory features suggests that the details of a sensory stimulus contribute to its perceived duration. Perceived duration is not only influenced by the property of sensory stimuli, but also by the history of stimuli: a repeated stimulus appears briefer than a novel stimulus (Tse et al., 2004; Pariyadath and Eagleman, 2007; Schindel et al., 2011; Birngruber et al., 2014). This phenomenon has been suggested to reflect a link between neural response amplitude and perceived duration (Pariyadath and Eagleman, 2007; Eagleman and Pariyadath, 2009). In addition, it was found that after adaptation to a fast drifting visual stimulus, a slow drifting visual stimulus is perceived as being of shorter duration when it appears at the adapted visual field, but not at other locations (Johnston et al., 2006; Ayhan et al., 2009, 2011; Bruno et al., 2010). The latter example not only highlights the involvement of low-level sensory processing in duration perception, but also demonstrates that stimuli in different parts of the visual field can provide different evidence of duration.

The finding that perceived duration can be biased by the sensory features of stimuli creates a puzzle. Even if visual objects at different locations last for the same physical duration, they each can bias perceived duration in different directions due to their sensory features. How does the brain form a representation of duration based on the duration estimates from different visual objects?

One possibility, as an extension of the hypothesis that perceived duration is based on neural response amplitude (Eagleman and Pariyadath, 2009), is that the perceived duration may be based on the sum of the total neural response to all the stimuli. An alternative hypothesis is that an estimate of duration is formed based on each stimulus and the brain integrates these estimates by a weighted average. A stronger statement of this hypothesis is that the integration may be statistically optimal (Ahrens and Sahani, 2011). A third hypothesis is that the brain may form a duration representation based on only one of the stimuli, with certain probability. A fourth hypothesis is that the brain may only rely on the stimulus type that provides more reliable (less variable) estimate of duration across trials. Lastly, it is possible that the brain may generate a representation of duration based on each stimulus and keep all the representations. In this last framework, the brain may have flexibility to choose which representation to use depending on the task.

Closely related to the question asked in this study, Ayhan et al. (2012) investigated whether human observers can average the durations of multiple objects. They flashed multiple images of different durations with asynchronous onsets and asked participants to make judgments with regards to the average duration of those images. The precision of the duration judgment was found to be worse when judging the average duration of multiple images than when judging the duration of a single image. The authors suggested that this reflects an inability to aggregate duration information from multiple items (Ayhan et al., 2012). While this may be the case when the stimuli have asynchronous onsets and offsets, there has been no study investigating whether and how human observers combine duration information from multiple objects which appear and disappear synchronously. To study the combination of duration information without introducing asynchrony between stimuli, we utilize the illusion that the temporal frequency of a visual stimulus biases perceived duration to create conflicting estimates of duration. In Experiment 1, we confirm this illusion by a two-alternative forced choice task. In Experiment 2, we qualitatively test the predictions of each of the above hypotheses to focus our attention on a few most plausible candidate models. In Experiment 3, we quantitatively compare these candidate models based on the trial-by-trial cross-validated log-likelihood of the models.

## Participants and methods

The experiments were approved by the Institutional Review Board of Baylor College of Medicine.

### Participants

Except for the first author, participants were all naïve to the purpose of the study. Participants provided informed consent and received compensation. Nineteen participants (8 males, 11 females. Age 27 ± 7) took part in Experiment 1. Twenty-one participants (13 males, 8 females. Age 29 ± 7) took part in Experiment 2. Twenty participants (6 males, 14 females. Age 27 ± 6) took part in Experiment 3.

### Apparatus

Experiment stimuli were displayed on a CRT monitor (Viewsonic G225f) with a screen resolution of 1024 × 768 pixels and a refresh rate of 100 Hz, driven by a Dell Precision T3400 workstation running Windows XP. There was no other light source other than the monitor in the experimental room. Participants sat at a distance of approximately 60 cm from the display. Each participant wore a pair of earplugs with approximately 33 dB noise reduction to prevent distraction.

### Stimuli

Stimuli were presented using Psychtoolbox 3 (Brainard, 1997; Pelli, 1997; Kleiner et al., 2007) for Matlab. Stimuli consisted of one or two drifting Gabor patches with spatial frequency of 0.28 cycle/degree (estimated at 60 cm viewing distance). The standard deviation of the 2-dimensional Gaussian envelop of each Gabor patch was 0.90°. The starting phase of each Gabor patch was independently sampled from a uniform distribution over the range of 0–2π. The peak luminance of the Gabor patch was 36.0 cd/m^2^. Stimuli were presented over gray background of mid-luminance. Each Gabor patch was displayed at a distance of 5.4° visual angle away from the fixation point. The fixation point was at the center of the screen, indicated by a white cross spanning a visual angle of 0.6°. Through the time course of each stimulus, the sinusoidal component of each Gabor patch drifted in a direction independently sampled from a uniform distribution over the range of 0–360°. The speed of their drifting was such that the luminance of any pixel of the Gabor patch was modulated by a sinusoidal time signal of either 1 Hz (for the low temporal frequency stimulus) or 6 Hz (for the high temporal frequency stimulus). At the onset of each stimulus, the contrast of the Gabor patch ramped up linearly from zero to maximum in 40 ms. At the offset, it ramped down in 40 ms. This ramping of the contrast was to minimize potential arousal introduced by abrupt onsets of stimuli.

Whenever two Gabor patches were displayed simultaneously, the centers of the two Gabor patches were on opposite sides from the fixation point, both on an invisible line that passed through the fixation point. In any trial, the orientation of the invisible line passing through the fixation point and the Gabor patch(es) in the first epoch was randomly sampled from a uniform distribution over 0–2π. The invisible line passing through the fixation point and the Gabor patch(es) in the second epoch was always orthogonal to the invisible line in the first epoch. This design was to minimize the effect of adaption due to presenting consecutive stimuli at the same location (Johnston et al., 2006).

### Experiment procedures

On each trial, a participant watched two groups of drifting Gabor patterns on the screen one after another and judged whether the duration of the second group was longer or shorter than that of the first group. Each group was composed of either a single Gabor patch drifting at 1 Hz (we denote this by L), or a single Gabor patch drifting at 6 Hz (we denote this by H), or a pair of Gabor patches, one at 1 Hz and the other at 6 Hz (we denote this by HL). In an HL stimulus, the two Gabor patches had the same onset time and offset time. The directions in which they drifted were randomly chosen and independent from each other. If a participant asked which one patch of the HL stimulus they should judge, he/she was instructed that since the patches appeared and disappeared synchronously, he/she should judge the duration in which both of them stay on the screen.

The structure of each trial was as follows. A trial started by a fixation cross appearing in the center of the screen. After a duration sampled from a uniform distribution over the range of 600–1000 ms, the first group of Gabor patch(es) appeared. 500–700 ms after the offset of the first group of Gabor patch(es), the second group appeared. 300–600 ms after the offset of the second group, the fixation cross disappeared and the participants were allowed to make response. They indicated the duration of the second group as lasting longer by pressing the right arrow key, or indicated it as lasting shorter by pressing the left arrow key. No feedback was provided. 1000–2000 ms after they made a response, the next trial started.

On any trial of an experiment, one group of Gabor patches lasted for 600 ms. We denote this stimulus of fixed duration by reference stimulus. The other group lasted for duration of one of 26 values between 100 and 1100 ms, equally spaced by steps of 40 ms. We denote this stimulus by comparison stimulus. For each of these 26 values, the number of its incidence was approximately proportional to the probability density of a Gaussian distribution with a mean of 600 ms and a standard deviation of 300 ms at that duration, rounded to the nearest integer. Thus, over the course of an experiment, the distribution of the duration of comparison stimuli approximates a truncated Gaussian distribution.

#### Experiment 1

There were two conditions in the experiment. In one condition, the reference stimulus was H and the comparison stimulus was L (denoted by LvsH). In the other condition, the reference was L and the comparison was H (denoted by HvsL). On half of the trials of each condition, the reference stimulus appeared before the comparison stimulus. On the other half of the trials, the comparison stimulus appeared before the reference stimulus. Each condition had 180 trials, including both orders of display. For each order of display in each condition, the comparison stimuli of 100, 140, 180, …, and 1100 ms occurred for 1, 2, 2, 2, 3, 3, 4, 4, 4, 5, 5, 5, 5, 5, 5, 5, 5, 4, 4, 4, 3, 3, 2, 2, 2, and 1 times. These numbers of incidences were generated to approximate a Gaussian distribution described above. Trials corresponding to different conditions, orders and comparison durations were randomly interleaved in a session. There was no signal to indicate to the participants which condition a trial belonged to.

#### Experiment 2

On all trials, the reference stimulus was an HL stimulus. The comparison stimulus was an L, H, or HL stimulus. The reference stimulus was always presented before the comparison stimulus. Each condition had 148 trials. In each condition, the comparison stimuli of 100, 140, 180, …, and 1100 ms occurred for 2, 2, 4, 4, 4, 6, 6, 6, 8, 8, 8, 8, 8, 8, 8, 8, 8, 8, 6, 6, 6, 4, 4, 4, 2, and 2 times. The trials of the three conditions were randomly interleaved.

#### Experiment 3

There were seven conditions in the experiment. In two conditions, the reference stimulus was H; the comparison stimulus was H or L, respectively. In two other conditions, the reference stimulus was L; the comparison stimulus was H or L, respectively. In the other three conditions, the reference stimulus was HL; the comparison stimulus was H, L, or HL, respectively. On half of the trials of each condition the reference stimulus was presented before the comparison stimulus. On the other half of the trials, the comparison stimulus was presented before the reference stimulus. Each condition had 228 trials. Each participant completed three sessions of experiment. For each order of display in each condition, the comparison stimuli of 100, 140, 180, …, and 1100 ms occurred for 3, 3, 3, 3, 3, 3, 3, 6, 6, 6, 6, 6, 6, 6, 6, 6, 6, 6, 6, 3, 3, 3, 3, 3, 3, and 3 times in total over all sessions. Trials corresponding to different conditions, orders and durations of comparison stimuli were randomly interleaved in a session. The number of trials corresponding to each condition, order and duration of comparison stimulus was equal across sessions.

## Results

### Experiment 1

It has been found that visual stimuli of higher temporal frequency or faster speed are perceived as lasting for longer than those of lower temporal frequency or slower speed (Kanai et al., 2006; Kaneko and Murakami, 2009). Our goal in Experiment 1 is to confirm this finding. In the previous literature, the overestimation of duration was measured by a reproduction task: after watching a stimulus, participants pressed a button for as long as they believed the stimulus had lasted. The variance of the reproduced duration in such a task is contributed to by the variance of participants' perceived duration and the noise in their motor timing. To avoid the latter, we used a two-alternative forced choice task, in which participants watched two consecutive stimuli and judged which lasted longer. This offers a more accurate estimation of the difference in perceived durations between stimuli of high and low temporal frequencies.

**Figure 1:**
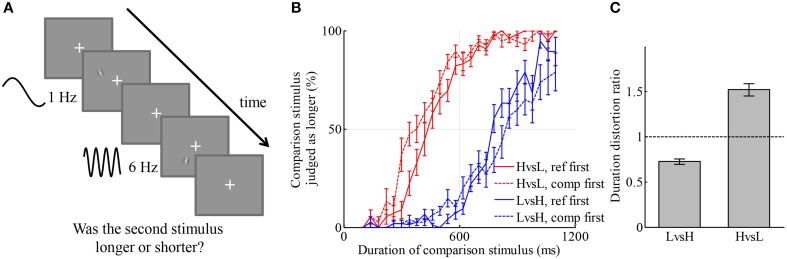
**Visual stimulus of higher temporal frequency is perceived as longer than that of lower temporal frequency. (A)** Illustration of an example trial. Two drifting Gabor patches with temporal frequencies of 1 Hz (low frequency) and 6 Hz (high frequency), respectively, were displayed consecutively with random order. One of them lasted for 600 ms (reference stimulus), the other lasted for a duration between 100 and 1100 ms (comparison stimulus). Participants judged which one stayed for a longer duration by pressing one of two keys. **(B)** Average psychometric curves of two conditions. Red color: the condition in which L was reference stimulus and H was comparison stimulus. Blue color: the condition in which H was reference stimulus and L was comparison stimulus. Solid lines: reference was displayed before comparison stimulus. Dashed lines: comparison stimulus was displayed before reference stimulus. **(C)** Duration distortion ratio of the comparison stimulus relative to the reference stimulus in the two conditions. High-temporal frequency stimuli were judged longer than low-temporal frequency stimuli.

The stimuli of an example trial are shown in Figure 1A. Each stimulus was a supra-threshold Gabor patch. Each pixel of the Gabor patch was modulated by a sinusoidal time series of either 1 Hz (we denote this low frequency by L) or 6 Hz (we denote this high frequency by H). Thus, the Gabor patch appeared as a grating that drifted behind a static 2-dimensional Gaussian aperture. The first Gabor patch appeared at a random location with fixed distance from the center of the screen (fixation point). The second Gabor patch appeared at the same distance from fixation but either 90° clockwise or counterclockwise from the first Gabor patch. On any trial, one of the stimuli lasted for 600 ms (we denote this as reference stimulus), and the other lasted for one of 26 durations equally spaced between 100 and 1100 ms (we denote this as comparison stimulus). The distribution of the duration of the comparison stimulus approximated a truncated Gaussian distribution with mean of 600 ms and standard deviation of 300 ms. On half of the trials, the comparison stimulus was H and the reference stimulus was L (HvsL condition). On the other half of the trials, the comparison stimulus was L and the reference stimulus was H (LvsH condition). On half the trials of each condition, the reference stimulus appeared before the comparison stimulus. On the other half, it appeared after. Participants reported whether the second stimulus lasted longer or shorter than the first stimulus.

The participant-averaged psychometric curves are displayed in Figure 1B. A leftward shift of a curve from centering at 600 ms indicates that the duration of the comparison stimulus was overestimated relative to the reference stimulus, and vice versa for a rightward shift. There was a slight discrepancy between the curves corresponding to different orders of display, namely, that curves deviated more from the reference duration and were shallower when the comparison stimulus was presented first. This type of discrepancy was also found in many other studies of perceptual judgments (Nachmias, 2006; Lapid et al., 2008; Bruno et al., 2010, 2012; Ahrens and Sahani, 2011). We will investigate the source of such discrepancy in Experiment 3, together with quantitatively comparing models of the representation of duration for simultaneously presented H and L stimuli. For simplicity, trials of different orders of display but belonging to the same condition were aggregated in the analysis. We fitted each participant's responses in each condition by a curve of Gaussian cumulative distribution on the logarithmic scale of duration, with an additional term capturing lapse rate, the chance that a participant had not paid attention to the stimuli (Wichmann and Hill, 2001). The ratio of the perceived duration of comparison stimuli to that of reference stimuli in each condition was calculated based on the exponential of the shift of the psychometric curve in the logarithmic scale. We denote this ratio by the duration distortion ratio (DDR, Figure 1C). In the LvsH condition, the duration of the L stimulus was judged as 27.3 ± 3.0% (mean ± s.e.m, the same through this paper unless otherwise stated) shorter than the H stimulus; the DDR was significantly smaller than 1 [*t*_(18)_ = −9.10, *p* < 0.001]. In the HvsL condition, the duration of the H stimulus was judged as 52.1 ± 6.8% longer than the L stimulus; the DDR was significantly larger than 1 [*t*_(18)_ = 7.67, *p* < 0.001]. The standard deviations of the fitted Gaussian cumulative distribution functions represent participants' sensitivity in discriminating duration in the two conditions, termed as just noticeable difference (JND). The JND was 0.27 ± 0.03 on the logarithmic scale of duration in the LvsH condition, and 0.35 ± 0.03 in the HvsL condition. They were significantly different [*t*_(18)_ = −3.99, *p* < 0.001]. The JND in logarithmic scale has similar meaning to Weber's ratio. When psychometric curves were fitted without applying logarithmic transformation of duration, the conclusions about DDR and Weber's ratio stayed the same. The absolute value of the DDR is very different between LvsH and HvsL conditions. This may indicate that the distortion in perceived duration caused by the temporal frequency is multiplicative instead of additive.

Experiment 1 confirms the previous finding that the perceived duration of visual stimulus is biased by its temporal frequency or speed. This leads to our main question: how do we perceive duration if two stimuli are presented simultaneously, one of which moves faster and the other slower. In Experiment 2, we test several hypotheses.

### Experiment 2

This experiment examined the perceived duration of two stimuli appearing simultaneously at different locations, one of low temporal frequency (L) and one of high temporal frequency (H). We denote such stimuli by HL. The H and L elements of it appear and disappear at the same time. This provides a clue that they should correspond to the same period of duration. However, following the observation in Experiment 1, the H and L elements of HL each should cause conflicting biases on the respective duration estimates, with H indicating a longer duration and L indicating a shorter duration. How does the brain form a representation of duration for the joint stimulus?

We consider five possibilities:

#### Global summing hypothesis

It is noticeable that neural response amplitude in visual cortex also increases with temporal frequency in the range that was tested in Kanai et al.'s experiments (Singh et al., 2000). The bias in perceived duration caused by the temporal frequency or speed of visual stimuli may be explained by assuming that perceived duration is based on the neural response amplitude to the stimulus (Eagleman and Pariyadath, 2009). It may also be explained by assuming that duration perception is based on the number of changes observed (Brown, 1995; Kanai et al., 2006). As possible extensions of both of these hypotheses, we may assume that the perceived duration of multiple elements is based on either the total neural responses to all the stimulus elements or the total number of changes in all stimulus elements. We denote such hypotheses by “global summing.” Both of them predict that HL should be perceived as lasting longer than both H and L.

#### Weighting hypothesis

The perceived duration of HL may be formed by a weighted average of each estimate of duration based on one of its elements. We denote by *x*_H_ the estimate of duration based on an H stimulus lasting for a physical duration of *t*, and denote by *x*_L_ the one based on an L stimulus lasting the same duration. *x*_H_ and *x*_L_ both vary across trials. We assume that their variations are independent and both follow Gaussian distributions:

(1) $$x_{\text{H}}\sim \text{N}(t+b_{\text{H}},\sigma_{\text{H}})$$

(2) $$x_{\text{L}}\sim \text{N}(t+b_{\text{L}},\sigma_{\text{L}})$$

*b*_H_ and *b*_L_ represent the bias of perceived duration introduced by their temporal frequencies. σ_H_ and σ_L_ represent the standard deviation of the distribution of *x*_H_ and *x*_L_. For simplicity, we assume that a point estimation of the duration of stimulus HL is formed by weighting *x*_H_ and *x*_L_:

(3) $$x_{\text{HL}}=w_{\text{H}}x_{\text{H}}\;+\;(1-w_{\text{H}})x_{\text{L}}$$

where the weight *w*_H_ is a parameter of each participant, in the range of [0, 1]. The distribution of *x*_HL_ would follow:

(4) $$x_{\text{HL}}\sim \text{N}(t\;+\;w_{\text{H}}b_{\text{H}}\;+\;(1-w_{\text{H}})b_{\text{L}},\sqrt{w_{\text{H}}^{2}\sigma_{\text{H}}^{2}\;+\;{\left(1-w_{\text{H}}\right)}^{2}\sigma_{\text{L}}^{2}})$$

For any weight *w*_H_, this predicts that on average HL is perceived equal to or shorter than H, and equal to or longer than L. The equality is only reached if *w*_H_ is 0 or 1, meaning one of the elements is neglected. It also predicts that the standard deviation of the perceived duration of HL is equal to or smaller than the larger one of those of H and L (namely, σ_HL_ ≤ max{σ_H_, σ_L_}). The equality is only reached when the duration estimation is only based on the more variable estimation between *x*_H_ and *x*_L_, i.e., when *w*_H_ = 1 and σ_H_ ≥ σ_L_, or when *w*_H_ = 0 and σ_H_ ≤ σ_L_.

The statistically optimal way to weight sensory evidence is by setting the weight of each duration estimation inversely proportional to the variance of that estimation (Jacobs, 1999; Knill and Pouget, 2004). We denote the hypothesis that the weighting follows this rule as the “optimal integration” hypothesis, as a stronger version of the “weighting” hypothesis. Based on this hypothesis, we expect the perceived duration of HL to be less variable than that of each stimulus element H and L:

(5) $$\sigma_{\text{HL}}=\sqrt{\frac{\sigma_{\text{H}}^{2}\sigma_{\text{L}}^{2}}{\sigma_{\text{H}}^{2}+\sigma_{\text{L}}^{2}}}\;<\text{ min}\{\sigma_{\text{H}},\sigma_{\text{L}}\}$$

#### Selection hypothesis

Instead of weighting the estimates based on the two stimulus elements, the brain may estimate the duration based on only one of the two elements. On some trials the perceived duration may be based on the H element and on other trials it is based on the L element. The element selected to form duration representation on a trial may be the one which more attention is paid to. Assuming a participant has a probability *c*_H_ to rely on the H element to estimate duration, we have

(6) $$x_{\text{HL}}=\{\begin{array}{l} x_{\text{H}},with\;probability\;c_{\text{H}}\\ x_{\text{L}},with\;probability\;(1-c_{\text{H}}) \end{array}$$

With the same notation as we used above, the mean of *x*_HL_ across trials would be

(7) $$t\;+\;c_{\text{H}}b_{\text{H}}+\left(1-c_{\text{H}}\right)b_{\text{L}}$$

and the standard deviation of *x*_HL_ across trials would be

(8) $$\sqrt{c_{\text{H}}\;\sigma_{\text{H}}^{2}\;+\;(1-c_{\text{H}})\;\sigma_{\text{L}}^{2}\;+\;c_{\text{H}}(1-c_{\text{H}}){\left(b_{\text{H}}\;-\;b_{\text{L}}\right)}^{2}}$$

This predicts that the average of the perceived duration of HL across trials is also equal to or shorter than that of H, and equal to or longer than that of L. Equality is only reached if *c*_H_ is equal to 0 or 1. As opposed to the “weighting” hypothesis, it predicts that the standard deviation of the perceived duration of HL across trials is equal or larger than the smaller one of those of H and L (namely, σ_HL_ ≥ min{σ_H_, σ_L_}). The equality is only reached when the duration representation is always based on the stimulus type which gives rise to a smaller variance of duration estimation, i.e., when *c*_H_ = 1 and σ_H_ < σ_L_, or when *c*_H_ = 0 and σ_H_ > σ_L_.

#### Reliable stimulus hypothesis

The brain might only rely on one of the stimulus types across all the trials, and the stimulus type it relies on may be the one that in general gives rise to more reliable estimation of duration. Under this hypothesis, if a participant estimates the duration of H with less variability than estimating the duration of L, the participant may always estimate the duration of HL based on the H element. If the participant estimates the duration of L with less variability, he/she may always rely on the L element to estimate the duration of HL. This hypothesis also predicts that σ_HL_ ≤ max{σ_H_, σ_L_}. The average perceived duration of HL may be shorter than that of H and longer than that of L across participants, if not all participants estimate a same type of stimulus between H and L more reliably than the other. However, for those who have more reliable estimates of duration based on H, the perceived duration of HL should be on average equal to that of H. And similarly for those who have more reliable estimates of duration based on L.

#### Multiple representations hypothesis

Instead of forming a single representation of duration as assumed by the above hypotheses, the brain might keep multiple representations of duration, each based on one of the two simultaneously presented stimuli. When asked to compare the duration of HL with the duration of a single stimulus, the brain might use one of the two representations formed during HL that is based on the stimulus element that is most similar to the single stimulus to be compared. For example, when viewing HL, the brain might keep one duration representation based on H and one based on L. When asked to compare the duration of HL with the duration of H, the brain might compare the representation based on the H element of HL with the duration representation of the single H stimulus. In this case, H should be judged to be of the same duration as HL on average. Similarly, L should also be judged equally long as HL. In other words, under this hypothesis, when the reference stimulus is HL and the comparison stimulus is H or L, the DDRs of H and L relative to HL should be equal.

**Figure 2:**
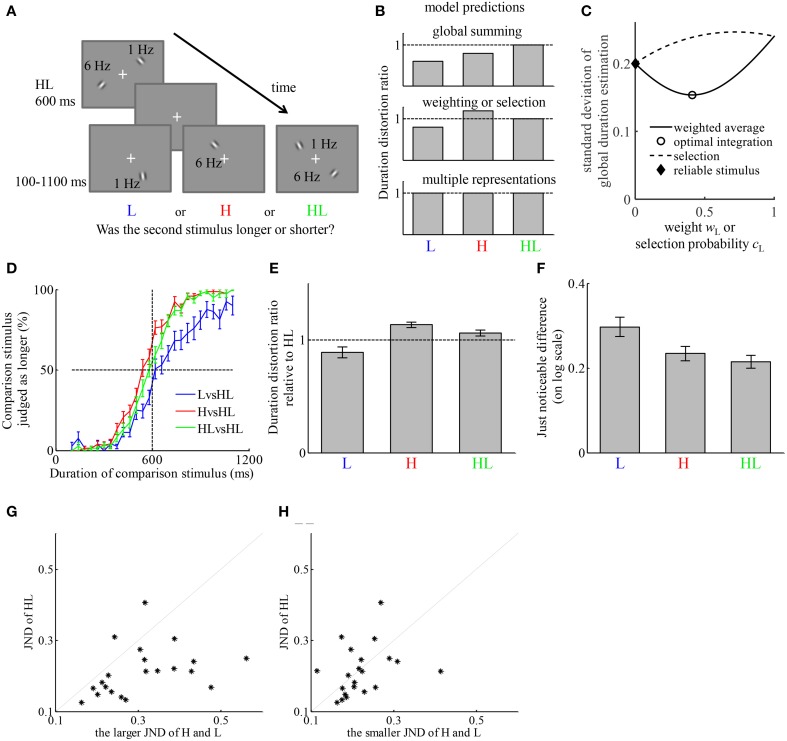
**The representation of duration of simultaneously presented high- and low-temporal frequency stimuli (HL) can be described by a weighted average of the estimates of duration based on the high-temporal frequency stimulus element (H) and low-temporal frequency stimulus element (L)**. **(A)** Example of the stimuli on a trial. Participants first viewed an HL stimulus lasting for 600 ms, then viewed one of three types of stimuli, H, L, or HL, with variable duration between 100 and 1100 ms. Participants judged which one lasted longer. **(B)** The qualitative relation between the duration distortion ratios of the comparison stimulus relative to the reference stimulus, predicted by four hypotheses of how the representation of the duration of HL is formed. The “reliable stimulus” hypothesis may generate the same prediction as “weighting” and “selection” hypotheses if not all participants estimate the same type of stimulus more reliably. **(C)** Illustration of the different predictions of the standard deviation of perceived duration of HL in comparison to that of H and L of the “weighting,” “optimal integration,” “selection,” and “reliable stimulus” hypotheses. The figure is generated by assuming σ_H_ = 0.2, σ_L_ = 0.24, and *b*_H_ – *b*_L_ = 0.2. **(D)** Average psychometric curves of the three conditions. **(E)** Average duration distortion ratio of the three conditions. **(F)** Average just noticeable difference (JND) of the three conditions. **(G)** Comparison between the JND in the HLvsHL condition and the larger JND of the other two conditions. Each dot corresponds to one participant. **(H)** Comparison between the JND in the HLvsHL condition and the smaller JND of the other two conditions.

To test the above predictions, we asked participants to compare the duration of H, L, or HL against the duration of HL. Example trials are shown in Figure 2A. On each trial, the reference stimulus was always presented before the comparison stimulus. The reference stimuli were all of HL type. There were three conditions distinguished by the types of comparison stimuli. In 1/3 of the trials, the comparison stimuli were L (LvsHL condition). In 1/3, the comparison stimuli were H (HvsHL condition). In the other 1/3, the comparison stimuli were HL (HLvsHL condition). Trials of the three conditions were randomly interleaved. Participants judged whether the duration of the second stimulus was longer or shorter than that of the first on each trial.

We tested the predictions of each of the models by comparing the DDRs between conditions. Each of the hypotheses generates prediction about the relation between the average perceived duration of HL and those of H and L. Figure 2B provides a qualitative illustration of their differences. The “weighting” and “selection” hypotheses generate the same qualitative prediction about the average perceived duration of HL. The “reliable stimulus” hypothesis may generate similar prediction as these two as long as there is individual difference regarding which of H and L is estimated with less variability. They are further distinguished by their qualitative predictions of σ_HL_, the standard deviation of perceived duration of HL. Without losing generality, by fixing the values of σ_H_, σ_L_ and *b*_H_-*b*_L_, Figure 2C illustrates how σ_HL_ varies as a function of *w*_H_ or *c*_H_, which are both free parameters of each participant. The “weighting” hypothesis predicts σ_HL_ ≤ max{σ_H,_ σ_L_} while the “selection” hypothesis predicts σ_HL_ ≥ min{σ_H,_ σ_L_}. Under the “optimal integration” hypothesis, a stronger version of the “weighting” hypothesis, we have σ_HL_ ≤ min{σ_H,_ σ_L_}. The “reliable stimulus” hypothesis predicts σ_HL_ ≤ max{σ_H,_ σ_L_}. The predictions about the average perceived duration of HL are tested by comparing the DDRs of each stimulus type relative to HL. Although the standard deviations of perceived duration of each stimulus type cannot be directly measured, they have monotonic relation with the JNDs in each condition. Therefore, the predictions about the standard deviations of perceived duration are tested by comparing the JNDs between conditions.

The participant-averaged psychometric curves are displayed in Figure 2D. We fitted each participant's responses similarly as in Experiment 1. The DDRs of the three conditions are displayed in Figure 2E. In the LvsHL condition, the duration of the L stimulus was judged as 11.0 ± 4.8% shorter than HL stimulus. In the HvsHL condition, the duration of the H stimulus was judged as 13.3 ± 2.5% longer than the HL stimulus. In the HLvsHL condition, the duration of HL as comparison stimulus was judged as 5.9 ± 2.7% longer than the HL as reference stimulus. A repeated measures ANOVA revealed a significant difference in DDR between the three conditions [*F*_(2, 40)_ = 11.81, *p* < 0.001]. *Post-hoc* paired *t*-tests between each two conditions revealed a significant difference between the LvsHL and HvsHL conditions [*t*_(20)_ = −4.21, *p* < 0.001], a significant difference between the LvsHL and HLvsHL conditions [*t*_(20)_ = −2.66, *p* = 0.015] and a significant difference between the HvsHL and HLvsHL conditions [*t*_(20)_ = 3.33, *p* = 0.003], all of which passed the Holm-Bonferroni multiple comparison criterion (Holm, 1979). The DDR in HvsHL condition was significantly larger than 1 (*t*-test, *p* < 0.001). The DDRs in the LvsHL was on average smaller than 1, but the difference was not significant after correcting for multiple comparison (*p* = 0.03, Holm–Bonferroni criterion). The DDR in the HLvsHL condition was also not significantly different from 1 (*p* = 0.04, Holm–Bonferroni criterion). The JNDs of the three conditions are shown in Figure 2F. Because the psychometric functions were fitted after logarithmic transformation of the duration, their units are also in the logarithmic scale. A repeated measures ANOVA revealed significant difference in JNDs between the three conditions [*F*_(2, 40)_ = 7.48, *p* = 0.002]. *Post-hoc* paired *t*-test between each pair of conditions revealed a significant difference between LvsHL and HvsHL conditions [*t*_(20)_ = 2.81, *p* = 0.011], a significant difference between the LvsHL and HLvsHL conditions [*t*_(20)_ = 3.57, *p* = 0.002], but no significant difference between the HvsHL and HLvsHL conditions [*t*_(20)_ = −0.02, *p* = 0.31]. The JND in the HLvsHL condition was significantly smaller than the maximum of those in the other two conditions [*t*_(20)_ = −4.23, *p* < 0.001], (Figure 2G) but not significantly different from the minimum of those in the other conditions [*t*_(20)_ = −0.40, *p* = 0.69] (Figure 2H).

The finding that HL was judged shorter than H argues against the “global summing” hypothesis. The “multiple representations” hypothesis is also ruled out because H and L was judged differently relative to HL stimulus. The pattern of DDRs among conditions of this experiment is consistent with both the “weighting” and “selection” hypotheses. The key difference of their predictions is with the standard deviation of the duration estimation of HL compared to those of H and L. JND indirectly reflects the standard deviation. The finding that JND in HLvsHL condition was smaller than the maximum of the JNDs in the other conditions supports the “weighting” and “reliable stimulus” hypotheses. The finding that it was not significantly different from the minimum of the JNDs in the other conditions does not provide support to the “selection” hypothesis or the “optimal integration” hypothesis. If the “reliable stimulus” hypothesis is true, then the participants who estimate the duration of H with less variability than L should have no difference in DDR between the HLvsHL and HvsHL conditions; the participants who estimate the duration of L with less variability should have no difference in DDR between the HLvsHL and LvsHL condition. Because the JND is smaller in HvsHL condition for majority of the participants (16 out of 21), we test the former prediction in these participants. The DDR was on average smaller in the HLvsHL condition (7.3 ± 3.2%) than in the HvsHL condition (12.5 ± 2.6%). The difference was marginally significant with *p* = 0.054.

We also note that the DDR in the HLvsHL condition was larger than 1, although the significance level did not pass our multiple comparison threshold. This may be due to participants' response bias or their prior belief about the relation between the first and second stimuli. However, such factors should equally impact all conditions. They do not influence our conclusions because the conclusions are based on comparisons between conditions. When psychometric curves were fitted without taking a logarithmic transform of duration, all conclusions remained the same except that the JNDs in LvsHL and HvsHL were not significantly different (*p* = 0.14), which was not crucial for testing the model predictions.

Therefore, the result of Experiment 2 provided qualitative evidence that the perceived duration of two dynamic stimuli is more likely formed by weighting the estimates of duration based on each individual stimulus, although we cannot entirely rule out the “reliable stimulus” hypothesis.

### Experiment 3

Experiment 2 ruled out the “global summing” and “multiple representations” hypotheses, provided qualitative support to the “weighting” hypothesis, but could not rule out the “reliable stimulus” hypothesis. The predictions of the “selection” and “optimal integration” hypotheses were not supported by the data, but they were also not entirely ruled out. In order to formally compare the “weighting” hypothesis, the “optimal integration” hypothesis, the “selection” hypothesis and the “reliable stimulus” hypothesis, one needs to explicitly model the decision process of each trial, predict the probability that a participant makes each judgment, and calculate the likelihood of each model. The probability that one stimulus is judged longer than another depends on both the mean and standard deviation of the perceived duration of the two stimuli over repetition of trials. As shown in Equations (4), (6), and (7), under each hypothesis, the mean and standard deviation of perceived duration of HL depends on those of the perceived durations of both H and L. Experiment 3 additionally included conditions in which the two stimuli on a trial were H and H, L and L, and H and L. These conditions constrained the fitting of parameters corresponding to the means and standard deviations of perceived duration of H and L, namely *b*_H_, *b*_L_, σ_H_, and σ_L_. In Experiment 1 we noticed a discrepancy in psychometric curves corresponding to different orders in which reference and comparison stimuli were displayed. To investigate the source of this discrepancy, trials of both orders of display were included for each condition in Experiment 3.

**Figure 3:**
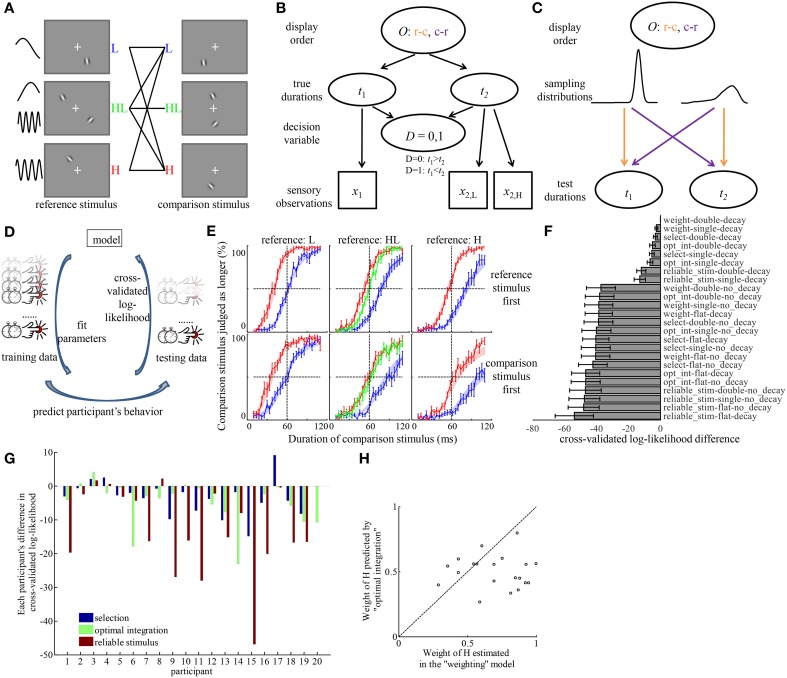
**Model comparison provides quantitative evidence for the “weighting” hypothesis and identified the source of the discrepancy in psychometric curves corresponding to different order of displaying reference and comparison stimuli. (A)** All the conditions tested in Experiment 3. Each condition corresponds to one solid line in the middle, connecting reference, and comparison stimuli. The order in which reference and comparison stimuli were displayed was random. **(B)** The generative model of an example trial for inferring the relation between two durations, if a participant considers the full structure of the task. *O*, order of display; c-r, comparison stimulus was displayed before reference stimulus; r-c, reference stimulus was displayed before comparison stimulus; *t*_1_, *t*_2_, durations of the first and second stimuli; *x*_1_, *x*_2_, sensory measurement of the first and second duration based on the stimuli; *x*_2, H_, *x*_2, L_, sensory measurements of the second duration, based on its H and L element, when the stimulus type is HL; *D*, decision variable indicating the relation between *t*_1_ and *t*_2_. **(C)** Illustration of how *O* decides the way *t*_1_ and *t*_2_ are sampled from two different distributions corresponding to the reference and comparison stimuli. The colors of the arrows correspond to the respective orders of display *O*. **(D)** The workflow of model comparison. Each model is fitted to part of a participant's trials (training data) to find the combination of parameters that maximized the probability of those trials. The fitted parameters are used to predict the behavior in the rest of the participant's trials (testing data). The probability of the testing data assuming the parameters fitted to the training data are logarithmically transformed to calculate the cross-validated log-likelihood. This procedure is repeated by rotating the selection of testing data over each of the 1/12 portion of the data. Models are compared based on the sum of cross-validated log-likelihood over all the data. **(E)** Average psychometric curves. Figures in the same column correspond to conditions of the same type of reference stimuli. Figures in the same row correspond to the same order of display. Color codes for the type of comparison stimuli. Shaded areas represent the fitted choice probabilities in each condition (mean ± s.e.m) by the best model in **(F)**. **(F)** The difference of cross-validated log-likelihood of each model compared to the best model. “weight,” weighting hypothesis; “select,” selection hypothesis; “opt_int,” optimal integration hypothesis; “reliable_stim,” reliable stimulus hypothesis; “flat,” flat prior hypothesis; “single,” single prior hypothesis; “double,” double priors hypothesis. **(G)** With individual variability, “weighting” model outperforms each of other models in most participants. The bars represent the differences of the cross-validated log-likelihood of the best models assuming each hypothesis regarding the mechanism of forming the representation of duration for HL stimulus, compared to that of the best model assuming “weighting” hypothesis. A negative bar indicates the model is inferior to the “weighting” model. Each group of bars corresponds to one participant. **(H)** Participants tended to overweight the duration estimate based on H stimulus. The coordinates of each dot correspond to the weight of H estimated in the “weighting” model and the weight of H predicted by the “optimal integration” model for each participant.

The timing structure of a trial in Experiment 3 was the same as in Experiment 1. There were seven conditions, defined by their reference and comparison stimuli. These conditions are illustrated in Figure 3A. The participant-averaged psychometric curves of each condition and each order of display are shown in Figure 3E. Similarly to Experiment 1, a discrepancy existed between the orders of displaying the reference and comparison stimuli. In general, psychometric curves were steeper and closer to the center of the range of duration when the reference stimulus was displayed first.

In order to understand the process of forming the representation of duration of HL and the discrepancy in judgments due to the order of display, we constructed models based on different hypotheses concerning three factors (van den Berg et al., 2014), and compared the log-likelihood of each model by cross-validating it within data of each participant. The details of the model comparison approach are described in Data Analysis and Modeling. Here we briefly list the major steps.

We consider the generative model of the sensory measurements of duration by the brain as in Figure 3B. The two durations to be compared on any trial were sampled from two distributions, one corresponding to the reference stimulus, and one corresponding to the comparison stimulus, as illustrated in Figures 3B,C. The order in which they were displayed was random from trial to trial. The true durations should be unknown to the brain. The brain only has sensory measurements of duration based on each of H or L stimulus, or each element of HL stimulus, which are noisy and biased by the temporal frequencies. We assume that the brain infers the relation between the two durations given its sensory measurements of duration from each stimulus or stimulus element. We further assume that the biases in sensory measurements are not accessible to the brain at the inference stage. It is very unlikely that the brain learns the true distributions from which the durations are sampled because of the noise in their sensory measurements and the biases introduced by different types of stimuli. For simplicity, we model the belief of the distributions by convolution of the true distributions of the durations (of reference and comparison stimuli) with a Gaussian kernel, as demonstrated in Figure 3C. The asymmetric shapes of these distributions result from the logarithmic transformation of duration.

We constructed models by all combinations of assumptions concerning each of three factors: how to form a representation of duration for HL, whether the memory of the sensory measurement of a stimulus' duration decays over time, and how the brain incorporates prior belief of the distributions of duration in their decision. After constructing these models, we performed a thorough factorial model comparison to examine the performance of each hypothesis in each of the three factors (van den Berg et al., 2014).

For the first factor, we considered the “weighting” hypothesis, “optimal integration” hypothesis, “selection” hypothesis, and “reliable stimulus” hypothesis. They differ in how the brain calculates the likelihood of any duration being the true duration, given the sensory measurements of duration based on each elements of HL.

For the second factor, we considered two hypotheses. Note that when participants made their judgments on any trial, more time had elapsed since the first stimulus than since the second stimulus. The first hypothesis, “decay” hypothesis, states that because of the elapse of time, the memory of the first duration decays more than the second, becoming noisier and more uncertain. To reflect this hypothesis, we assumed that the standard deviation of the sensory measurement of the first duration is scaled up by a constant factor relative to that of the second duration. The second, “no decay” hypothesis, states that the standard deviation is the same regardless of whether a stimulus is presented first or second.

For the third factor, we considered three hypotheses. The first one, the “flat prior” hypothesis, states that the brain does not take into account any prior distribution of duration, thus its judgments are purely based on sensory measurements of duration. The second one, the “single prior” hypothesis, states that the brain learns the mixture of the durations of reference and comparison stimuli as a global distribution and assumes that both durations on any trial are sampled from this distribution. The third one, the “double priors” hypothesis, states that the brain learns the full structure of the generative model in Figure 3C that the two durations on any trial are sampled from two different distributions and displayed in random order. Consequently, it incorporates the two learnt distributions and considers both the possible orders of display in the decision process.

The workflow of the model comparison is illustrated in Figure 3D. For each model, we derived the decision rules of judging the relation between two durations given any possible sensory measurements on a trial. By integrating the hypothesized distributions of sensory measurements over the range where one of the two judgments should be made according to the decision rule, we obtained the probability that a participant should have made that judgment on any trial (we denote this by choice probability). The choice probability depends on the parameters in each model. Each model thus can be fitted to a subset of data (denoted by training data) of a participant by finding the parameters that maximizes the product of the choice probabilities of all trials in the training data. Each model can be evaluated by predicting the probabilities of the judgments that the participant had made in the rest of the trials (denoted by testing data) based on the parameters fitted to the training data. We conducted 12-fold cross-validation of each model on each participant's data. The logarithm of the product of predicted probabilities over all testing data in the 12-fold cross-validation was compared between models. We denote this measure by cross-validated log-likelihood. This measure is not sensitive to the complexity of the models. A model that is unnecessarily complex would be overfitted to the training data, resulting in low cross-validated log-likelihood.

Figure 3F shows the difference of cross-validated log-likelihood of each model from the model that is on average the best across all participants. The more negative the difference is, the worse a model performs. There are several observations from this figure. (1) The largest distinction of model performance was introduced by the assumptions about memory decay and prior belief of duration distribution. Models that assume the existence of memory decay and assume the brain incorporates prior belief of the duration distribution in either form of “single prior” and “double priors” largely outperformed models that do not make these assumptions. By investigating the choice probability predicted by each model, we found that only the combination of the assumptions of memory decay and incorporation of prior(s) of non-flat form can introduce a difference in choice probability between different orders of displaying reference and comparison stimuli. (2) On average across participants, the “weighting” hypothesis was the best model to describe the representation of duration of the HL stimulus. Among models that can explain the effect of displaying order, the best model was the one assuming a combination of the “weighting” hypothesis, the “decay” hypothesis and the “double priors” hypothesis in the three factors, respectively. Paired *t*-tests between the cross-validated log-likelihood of all other models and that of the best model revealed that the best model outperformed every of other models significantly (The *p*-values passed Holm–Bonferroni multiple comparison thresholds with α = 0.05. The largest *p*-value was 0.016 when comparing the best model against the model assuming a combination of “optimal integration,” “decay,” and “double priors”). The average difference across participants between the best model and the models with other hypotheses regarding the representation of the duration of HL was at least 3.2 (the best among those models with other hypotheses was the one assuming “selection,” “decay,” and “double priors”). Notice that this difference is in the logarithmic scale. It means that the best model with the “weighting” hypothesis performs at least 25 times as well as models assuming other hypotheses regarding the perceived duration of HL. Since the cross-validated log-likelihood is on the same scale as Bayes factor, the guidance of drawing conclusion on model performance based on Bayes factor (Kass and Raftery, 1995) can help judge the strength of evidence for the best model. According to Kass and Raftery, such difference as observed in the result of Experiment 3 is considered as “strong” evidence for the best model. Figure 3E overlays the average psychometric curves over the choice probability fitted by the best model.

Figure 3G displays the model performance for each individual participant, focusing on the mechanism of estimating duration of HL. For each participant and for each hypothesis regarding the perceived duration of HL, we identified the best model among the ones with that hypothesis. The difference in cross-validated log-likelihood between each of these best models and the best model with the “weighting” hypothesis is plotted in Figure 3G for each participant. Although there is individual difference with respect to the best model for each participant, the “weighting” hypothesis outperforms each of other hypotheses in most participants.

We further compared the estimated weight of H element in the best model with the weight predicted by “optimal integration” based on the standard deviation of the duration estimates of the H and L (Figure 3H). The participants' weights of H element (0.70 ± 0.05) were significantly larger than those predicted by “optimal integration” [0.50 ± 0.03, paired *t*-test, *t*_(19)_ = 3.53, *p* = 0.002]. There was no significant correlation between weights estimated in the best model and the weights predicted by “optimal integration” (*p* = 0.86).

**Figure 4:**
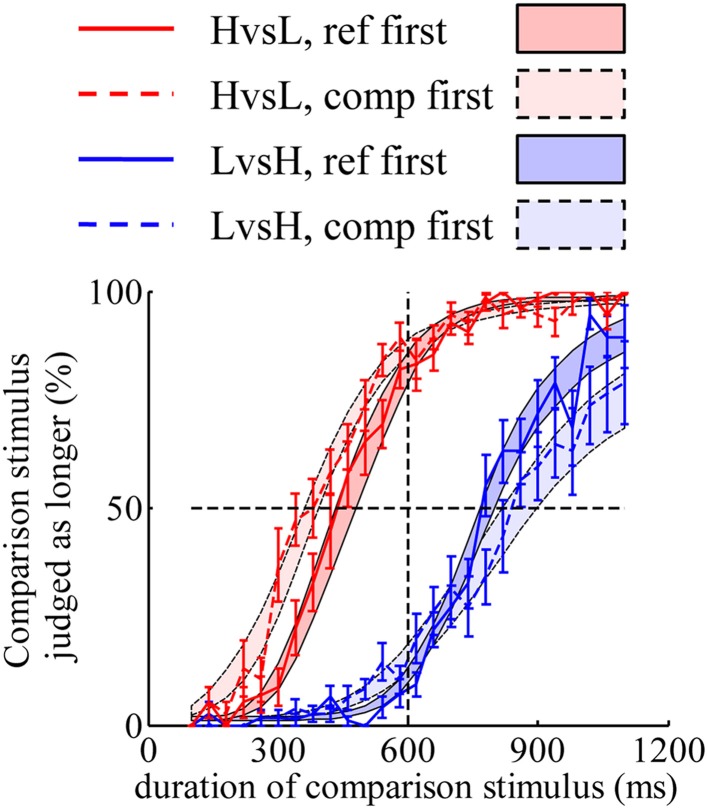
**A model constructed with “decay” and “double-priors” hypotheses captures the discrepancy in psychometric curves observed in Experiment 1**. Shaded areas represent the predicted choice probabilities.

The discrepancy in psychometric curves found in Experiment 1 can also be accounted for by the same mechanism found in Experiment 3. A model constructed with “decay” and “double-priors” hypotheses fitted well to the psychometric curves (Figure 4). Models constructed with “no-decay” or “flat-prior” hypotheses cannot predict such discrepancy corresponding to different orders of display (figures not shown).

The result of Experiment 3 confirmed that the representation of duration of HL is best described by weighting the duration estimates based on each stimulus element. The brain appears to weight H more than predicted by “optimal integration.” In addition, it shed light on the source of discrepancy in participants' judgments between different orders of displaying reference and comparison stimuli. Degradation of memory with elapsing time and incorporation of prior distributions of duration jointly account for this discrepancy.

## Discussion

In this study, we first used a two-alternative forced choice task to confirm previous finding that perceived duration is biased by the temporal frequency or speed of a visual stimulus. We further asked how the brain forms a representation of duration when two visual stimuli are displayed simultaneously, one of lower temporal frequency and one of higher temporal frequency. By both qualitatively testing predictions of different models and quantitatively comparing models based on cross-validated log-likelihood, we concluded that the model that best explains the data assumes the duration representation of such joint stimuli is formed by weighting the estimates of duration based on each stimulus element. However, participants' behavior could not be explained well by the framework of statistically optimal integration. Instead, they tended to overweight the evidence of duration from the stimulus element of higher temporal frequency. In addition, we found that the joint effect of memory decay and incorporation of prior belief of the distributions of duration can account for a discrepancy between psychometric curves of trials belonging to the same condition but with different orders of displaying reference and comparison stimuli.

Previously, the perceived duration of a sequentially concatenated stimulus that is composed of intermittent periods of static and drifting stimuli was found to be perceived shorter than a constantly drifting stimulus of the same duration, but not different from a static stimulus (Bruno et al., 2012). This appears in contrast to our finding that participants overweight the estimate based on the H element when estimating the duration of HL. We should note that in their experiment, the static and drifting intervals of a stimuli were concatenated, rather than presented simultaneously. Therefore, estimating duration of the concatenated stimulus may be viewed as summing the durations of each short interval during which the stimulus was constantly drifting or static instead of averaging the durations of those short intervals. In contrast, the H and L elements in our HL stimulus were displayed simultaneously. Given the large difference in the temporal structures of the stimuli between the two studies, the results of the two studies may not be directly comparable.

In all of our analyses, the curve fitting and modeling were performed after taking logarithmic transformation of duration. This was done because the Weber's law in duration perception (Gibbon, 1977; Buhusi and Meck, 2005) can be easily captured by assuming a constant level of noise on a logarithmic scale of duration. Fitting a Gaussian cumulative function to the data in Experiment 1 and 2 without logarithmic transformation generated qualitative identical results in all the comparisons critical to our conclusions. We did not attempt to model the data of Experiment 3 on a linear scale of duration because the assumption that sensory measurements follow a Gaussian distribution on a linear scale would result in negative duration estimates, which is meaningless. Additional complexity exists if one chooses to model in linear scale and to assume that the standard deviation of the sensory measurement scales with the duration, because the likelihood function cannot be analytically described by Gaussian function anymore in such a case (Girshick et al., 2011).

In our experiments, we utilized the illusory phenomenon that perceived duration is biased by the temporal frequency or speed of a visual stimulus (Kanai et al., 2006; Kaneko and Murakami, 2009) to manipulate the length of perceived duration without changing the physical duration of a stimulus. There still exists a debate on whether the bias is induced by temporal frequency or speed (Kaneko and Murakami, 2009; Linares and Gorea, 2015). Our result is independent from the answer to this debate, because the spatial frequency was constant in all stimuli and temporal frequency was proportional to speed in our experiments. One may worry that observers could have just used the onsets and offsets to judge duration in our task. This possibility is not compatible to our result because purely judging duration based on the onsets and offsets would not give rise to the difference in perceived duration between H and L, or between HL and the other two types of stimuli.

Several hypotheses have been proposed to account for the influence of temporal frequency or speed on perceived duration. Our results may provide constraints to these hypotheses. First, one hypothesis was that perceived duration may be based on the amount of change in the environment (Fraisse, 1963; Gibson, 1975; Poynter, 1989; Brown, 1995; Kanai et al., 2006). A quantitative formalization of this idea in the Bayesian observer framework was recently introduced (Ahrens and Sahani, 2011). A second hypothesis was based on the observation that stimuli of longer perceived duration, including those of higher temporal frequencies, typically also elicit larger neural responses. This hypothesis proposed that perceived duration may reflect the neural energy expended to encode sensory stimuli (Pariyadath and Eagleman, 2007; Eagleman and Pariyadath, 2009). Lastly, within the traditional “internal clock” framework of time perception, another hypothesis proposed that fluctuation of neural activity in visual cortex modulated by sensory stimuli may play a role in the tick rate of the clock (Kanai et al., 2006; Kaneko and Murakami, 2009). For the hypothesis based on amount of changes, our results suggest that perceived duration is not based on the total number of changes in all stimuli. Similarly, for the hypothesis based on neural energy, our results suggest that the perceived duration is not formed by summing the neural response to all stimuli, at least for dynamic stimuli. Both of these hypotheses can still be valid if we assume that duration estimates are based on local stimuli and these estimates are further weighted to form a global representation. For the hypothesis within an “internal clock” framework, our results suggest that the clock signals may come from distributed sources in sensory cortex and the tick counts from each source may be fused by weighted average. In contrast, if one assumed there is only one centralized clock, it would be difficult to explain the difference in JNDs when participants compare different types of stimuli. Although our “weighting” hypothesis resembles the spirit of cue integration in the Bayesian observer model, the “optimal integration” hypothesis did not provide the best account for our data.

Note that our implementation of the “optimal integration” hypothesis in Experiment 3 made some simplifying assumptions compared to the modeling framework of Ahrens and Sahani (2011). First, in their paper, the likelihood of duration was calculated as the probability of observing the changes between several samples in a dynamic luminance signal by assuming the signal follows the temporal statistics in natural scenes. By simulating this calculation one can obtain the biases of perceived duration due to different temporal frequencies. We did not use this approach to predict the biases because we found that the bias depends on free parameters such as the number of samples, sampling rates, and the contrast of stimuli compared to that of luminance signals in natural scene. Instead, we simply assumed the biases and standard deviations of the sensory measurements of duration are free parameters for each participant. This simplification should not influence our conclusion as long as the distribution of sensory measurements predicted by simulating their model approximates a Gaussian distribution. Second, in the model of Ahrens and Sahani's, there was an additional source of duration estimation purely based on internal neural activity, independent from the sensory inputs. We did not include this internal estimation in our models because it was shown that this internal estimation was not crucial to the predictions of their model (Ahrens and Sahani, 2011). However, even if we had included such an internal estimation, optimal integration should still predict σ_HL_ ≤ min{σ_H_, σ_L_} in Experiment 2, which was not reflected in the comparison of JNDs.

In Experiment 3, we found that memory decay and incorporation of the prior distributions of duration together account for the discrepancy in the threshold and slope of psychometric curves corresponding to different orders of display. The discrepancy in threshold resembles a phenomenon sometimes called the “time-order error” (Hellstrom, 1985). A similar discrepancy in the slope of psychometric curves was also found in many other studies of perceptual judgments (Nachmias, 2006; Lapid et al., 2008; Bruno et al., 2010, 2012; Ahrens and Sahani, 2011). It was proposed that an implicit standard was used in such comparison (Nachmias, 2006; Lapid et al., 2008). In our minds, this so-called “implicit standard” or “internal standard” plays a similar role as the prior distribution in our “single prior” model. In the model by Lapid et al. (2008), participants only weight the “internal standard” with the sensory evidence of the first stimulus but not with that of the second stimulus. In our models assuming “single prior” and “memory decay,” the decay of memory causes the likelihood function of the first duration to be wider than that of the second. This in turn makes the influence of the prior distribution to the posterior distribution for the first duration stronger than for the second. This is similar to giving more weight to the “internal standard” when calculating a weighted average of the “internal standard” and the sensory estimate of duration. Our modeling result (Figure 3F) suggests that such discrepancy due to the order of display may reflect an optimal strategy to integrate sensory evidence with prior belief of the structure of the task. A similar model was recently proposed to account for an order effect in a task of discriminating lengths of bars (Ashourian and Loewenstein, 2011). The fact that a common mechanism can account for related phenomena in both spatial and timing tasks indicates that similar inference strategies may be used in various domains of perceptual tasks. Here we give an intuitive explanation of why the prior distributions and memory decay jointly causes the effect of the displaying order, taking the “double priors” hypothesis as an example. Under this hypothesis, the brain separately calculates the posterior probabilities of the first duration being longer/shorter than the second based on each hypothetic order of display, and averages these probabilities to make the final judgment. To calculate the posterior probabilities of the relation between the durations, the brain needs to calculate the posterior probabilities of the duration of each stimulus. The prior distribution learnt from the comparison durations is much flatter than that learnt from the standard duration, and is thus less informative. Because it is less informative, it has smaller contribution to the posterior distribution no matter if it is used to infer the duration of a standard stimulus or of a comparison stimulus. On the contrary, the prior distribution corresponding to the standard duration is more concentrated and thus more informative. But it is only beneficial to the accuracy of judgment when it is used to calculate the posterior distribution of the duration for a stimulus that is actually the standard stimulus. If it is used to calculate the posterior distribution of a comparison stimulus, it “drags” the mass of the posterior distribution toward the standard duration, which makes the judgment more difficult. On the other hand, the relative contribution of the prior distribution to the posterior distribution also depends on the shape of the likelihood function of duration. The prior has relatively stronger impact on the posterior if the likelihood is flatter (less informative). This is the case for a stimulus that appears first in a trial, due to the decay of memory. Therefore, in the trials of which the first stimulus is the standard stimulus, the prior distribution corresponding to the standard duration provides larger benefit for estimating the posterior distribution of the standard duration but generates less “dragging” effect on the posterior distribution of the comparison stimulus. In the trials of which the first stimulus is the comparison stimulus, the “dragging” effect is stronger for the comparison stimulus but the benefit is weaker for the standard stimulus. This explains why the psychometric curve is steeper when the standard stimulus appears first.

One may worry that the order effect may be caused by lower uncertainty of the location of the second stimulus than that of the first. Because the effect of the order of display is observed in many other studies which do not manipulate the location of stimuli as we do, we think the difference in uncertainty of the position of the stimuli is unlikely the major cause of the order effect.

Observers' behavior in cross-modality cue combination tasks of many spatial features can often be well described by statistically optimal integration or appear close to optimality (Jacobs, 1999; Ernst and Banks, 2002; Battaglia et al., 2003). However, it is puzzling that behavior in cue combination tasks of duration or other temporal features often deviates from optimality in one way or another (Burr et al., 2009; Shi et al., 2010; Hartcher-O'Brien and Alais, 2011; Tomassini et al., 2011). Are brains simply suboptimal when it comes to time? It is difficult to give a comprehensive explanation of the sub-optimality; we can only provide some speculations. The first possibility is the role of causal inference (Knill, 2003, 2007; Körding et al., 2007; Shams and Beierholm, 2010): the brain not only needs to integrate different cues to form a more reliable estimation, but also needs to infer which of the cues may be generated by a different cause and should not be integrated. When two cues conflict too much or their relation violates some constraints, the brain should not integrate them but should instead treat them as from different sources. In spatial cue integration tasks, the temporal contingency between cues provides a strong clue that the cues may be generated from the same source. Unfortunately, in order to study duration cue combination, researchers often have to make the physical durations of the stimuli different (Hartcher-O'Brien and Alais, 2011; Ayhan et al., 2012). This creates asynchrony in onset and offset time between stimuli, which provides a strong clue that they should not be integrated. In fact, Ayhan et al. (2012) found a poorer performance when judging the average duration of multiple asynchronous stimuli than when judging the duration of a single item. They also found no significant difference between judging two items and judging eight items. It is possible that when stimuli are asynchronous, the brain does not perform weighted average but randomly selects one stimulus to estimate duration. Our use of temporal frequency to bias perceived duration avoided this asynchrony. However, it is still possible that the difference between the duration estimates of the H and L elements may be too large for participants to integrate them on some trials. Future studies that systematically manipulate the temporal frequencies of the two stimuli may help answer whether causal inference is the major cause of the apparent sub-optimality in combining duration estimates. A second possibility is that the stimuli used are not common in the natural environment and the brain may have a wrong belief about the precision of duration estimation based on each type of stimulus. Third, the H element may draw more attention than the L element, and the reliability of duration estimation may be changed due to different levels of attention. Lastly, it is possible that participants may have insufficient knowledge of some task-relevant information. For example, they may have learnt a wrong prior distribution, which may translate to apparent sub-optimality. These possibilities all call for future investigation. We believe that our approach of manipulating perceived duration can be further extended in studying many questions related to the integration of duration estimation.

In our experiments, we only manipulated the bias of perceived duration by temporal frequency, but did not attempt to manipulate the precision of the perceived duration. The difference in the precision of duration estimates of H and L were inherent to each participant. This reflects another limitation in studying cue combination in time perception: to our knowledge, there are few, if any, manipulations of visual stimuli that can independently influence the magnitude and precision of perceived duration (although see Hartcher-O'Brien et al., 2014, where the precision of perceived duration of auditory stimuli was manipulated by the signal to noise ratio of a tone). It is still largely unknown what determines the precision of duration estimation of different types of stimuli, such as the H and L stimuli in our experiments. Understanding how and why variability of duration perception changes with different stimulus features may provide insights into the mechanism by which duration is estimated based on sensory signals. Quantifying the statistics of natural scenes and deriving the optimal encoding and decoding strategy has been a fruitful approach in generating models for how the brain might solve spatial perception tasks. The performances of such models often highly resemble the performance of human observers (Geisler et al., 2009; D'Antona et al., 2013; Burge and Geisler, 2014). Only a few studies in time perception have taken this perspective (Ahrens and Sahani, 2011). We speculate that further analysis of the statistical structure of temporal signals in natural environments may identify the optimal strategy to estimate time based on natural signals and provide ways to understand the variability in duration judgments.

## Data analysis and modeling

### Experiment 1

We fitted each participant' responses by psychometric functions with shapes following Gaussian cumulative distribution. Trials of both orders of display belonging to the same condition were treated equally when fitting a psychometric function to them.

For trials in the LvsH condition, we denote by *t*_i, L_ the logarithmic transformation of the physical duration of the comparison stimulus on the *i*th trial. Similarly, for trials in the HvsL condition, we denote by *t*_i, H_ the logarithmic transformation of the physical duration of the comparison stimulus on the *i*th trial. We assume that the probability of a participant's response *r*_i, L_ for the *i*th trial of the LvsH condition is

(9) $$\begin{array}{l} p\left(r_{i,\text{L}}="\text{longer}"|t_{i,\text{L}},b_{\text{LvsH}},\sigma_{\text{LvsH}},\lambda \right)\\ =\left(1-\lambda \right)\Phi \left(t_{i,\text{L}}+b_{\text{LvsH}};t_{\text{ref}},\sigma_{\text{LvsH}}\right)+\frac{1}{2}\lambda \end{array}$$

(10) $$\begin{array}{l} p\left(r_{i,\text{L}}\;="\text{shorter}"|t_{i,\text{L}},b_{\text{LvsH}},\sigma_{\text{LvsH}},\lambda \right)\\ =1-p\left(r_{i,\text{L}}\;="\text{longer}"|t_{i,\text{L}},b_{\text{LvsH}},\sigma_{\text{LvsH}},\lambda \right) \end{array}$$

Similarly, we assume the probability of response *r*_*i*, H_ for the *i*th trial of HvsL condition is

(11) $$\begin{array}{l} \;p\left(r_{i,\text{H}}="\text{longer}"|t_{i,\text{H}},b_{\text{HvsL}},\sigma_{\text{HvsL}},\lambda \right)\\ =\left(1-\lambda \right)\Phi \left(t_{i,\text{H}}\;+\;b_{\text{HvsL}};t_{\text{ref}},\sigma_{\text{HvsL}}\right)\;+\;\frac{1}{2}\lambda \end{array}$$

(12) $$\begin{array}{l} \;p\left(r_{i,\text{H}}="\text{shorter}"|t_{i,\text{H}},b_{\text{LvsH}},\sigma_{\text{HvsL}},\lambda \right)\\ =1-p\left(r_{i,\text{H}}="\text{longer}"|t_{i,\text{H}},b_{\text{HvsL}},\sigma_{\text{HvsL}},\lambda \right) \end{array}$$

where λ is the probability that the participant would make random guess (lapse rate, common for both conditions); *b*_LvsH_ is the bias of perceived duration of stimulus L relative to H in the LvsH condition (in the log scale of duration); *b*_HvsL_ is the bias of perceived duration of stimulus H relative to L in the HvsL condition; σ_LvsH_ and σ_HvsL_ reflect the sensitivity to duration difference in the two conditions (JND on the logarithmic scale of duration). Φ(·) is Gaussian cumulative distribution function.

We assumed the responses are independent between trials.

The likelihood of the parameters L (*b*_LvsH_, σ_LvsH_, *b*_HvsL_, σ_HvsL_, λ) = *p*(data |*b*_LvsH_, σ_LvsH_, *b*_HvsL_, σ_HvsL_, λ) could then be calculated by the product of the probability of response for each trial:

(13) $$\begin{array}{l} \text{ L}\left(b_{\text{LvsH}},\sigma_{\text{LvsH}},b_{\text{HvsL}},\sigma_{\text{HvsL}},\lambda \right)\\ =p\left(\text{data}|b_{\text{LvsH}},\sigma_{\text{LvsH}},b_{\text{HvsL}},\sigma_{\text{HvsL}},\lambda \right)\\ =\prod_{i=1}^{N}\text{P}\left(r_{i,\text{L}}|t_{i,\text{L}},b_{\text{LvsH}},\sigma_{\text{LvsH}},\lambda \right)\cdot \\ \;\prod_{i=1}^{N}\text{P}\left(r_{i,\text{H}}|t_{i,\text{H}},b_{\text{HvsL}},\sigma_{\text{HvsL}},\lambda \right) \end{array}$$

where N is the number of trials in each condition. For each participant, we fitted all the parameters *b*_LvsH_, *b*_HvsL_, σ_LvsH_, σ_HvsL_, and λ simultaneously to maximize L(*b*_LvsH_, σ_LvsH_, *b*_HvsL_, σ_HvsL_, λ), using the “fmincon” function in Matlab. Since the curve fitting was performed after logarithmic transformation of duration, the bias terms *b*_LvsH_ and *b*_HvsL_ represent duration distortion in the logarithmic scale. We then calculated *e*^*b*^^LvsH^ and *e*^*b*^^HvsL^ as the duration distortion ratio plotted in Figure 1C.

### Experiment 2

The procedure of fitting parameters of psychometric functions was similar to that in Experiment 1. The bias terms *b*_LvsH_ and *b*_HvsL_ were replaced by *b*_L_, *b*_H_, and *b*_HL_, corresponding to the bias of the perceived duration of each type of comparison stimulus relative to that of the reference stimulus (in the log scale of duration). The JND terms σ_LvsH_ and σ_HvsL_ were replaced by σ_L_, σ_H_, and σ_HL_ for each condition.

### Experiment 3

#### Generative model

Participants' judgments were considered as an inference process. In Figure 3B, we illustrate an example of the generative models which we assume this inference process may be based on if the brain considers the full structure of the task. On each trial, a binary variable *O* determines the order in which the stimuli of different durations are displayed to the participant. With probability of 0.5, the reference stimulus is displayed before the comparison stimulus (we denote this by *O* = “r-c”). With probability of 0.5, the comparison stimulus is displayed before the reference stimulus (we denote this by *O* = “c-r”). *t*_1_, the true duration of the first stimulus, and *t*_2_, the true duration of the second stimulus, are sampled from the corresponding distributions of reference stimulus and comparison stimulus. Figure 3C illustrates this sampling process. The brain does not have access to the order *O* or the true durations *t*_1_ and *t*_2_. Instead, it has noisy neural measurements of durations that can vary from trial to trial. We denote these measurements by *x*_1_ and *x*_2_. Here, *t* and *x* are both in logarithmic scale of duration.

In the cases that the stimulus type in duration *t*_*i*_(*i* = 1, 2) is H or L, we assumed that the distribution of *x*_i_ follows a Gaussian distribution on the logarithmic scale of duration. The mean of the distribution is biased by the corresponding stimulus type H or L, as described in Equations (1) and (2).

In the case that the stimulus type in duration *t*_*i*_ (*i* = 1, 2) is HL, one noisy measurement is generated based on each element of HL. Figure 3B illustrates an example of such a case when the stimulus of duration *t*_2_ is HL. We denote the measurements based on the two elements of HL by *x*_2_ = {*x*_2, H_, *x*_2, L_}. We assumed that the distribution of duration measurement based on each element is the same as when only that element is displayed, and independent from each other:

(14) $$x_{i,\text{H}}\sim \text{N}(t\;+\;b_{\text{H}},\sigma_{\text{H}}^{2})\;(i=1,2)$$

(15) $$x_{i,\text{L}}\sim \text{N}(t\;+\;b_{\text{L}},\sigma_{\text{L}}^{2})\;(i=1,2)$$

#### Inference process

The brain only has access to *x*_1_ and *x*_2_. What participants report is their belief of the relation between *t*_1_ and *t*_2_, denoted by decision variable *D* (*D* = 0 means *t*_1_ > *t*_2_ and *D* = 1 means *t*_1_ < *t*_2_). The process of generating a response about *D* based on noisy observations *x*_1_ and *x*_2_ is the inference process that we modeled.

We assumed that the brain estimates the posterior distributions of stimulus durations *t*_1_ and *t*_2_ based on *x*_1_ and *x*_2_:

(16) $$p(t_{i}|x_{i})\;=\frac{p(x_{i}|t_{i})\cdot p(t_{i})}{p(x_{i})},\;(i=1,2)$$

The posterior distribution is proportional to two factors: *p*(*t*_*i*_), the prior distribution of *t*_*i*_, and *p*(*x*_*i*_ |*t*_*i*_), the likelihood of *t*_*i*_. The former is a participant's belief of the general distribution of the duration in the experiment without any sensory evidence. The latter is the probability that any particular *t*_*i*_ can generate the sensory measurement *x*_*i*_, regardless of the prior belief.

Based on *p*(*t*_*i*_ |*x*_*i*_), the brain further calculates the posterior probability of the decision variable *D*:

(17) $$\begin{array}{l} \;p\left(D=0|x_{1},x_{2}\right)=p\left(t_{1}>t_{2}|x_{1},x_{2}\right)\\ =\int_{-\infty }^{+\infty }dt_{1}\int_{-\infty }^{t_{1}}dt_{2}p\left(t_{1}|x_{1}\right)p\left(t_{2}|x_{2}\right) \end{array}$$

(18) $$\begin{array}{l} \;p\left(D\;=\;1|x_{1},x_{2}\right)=p\left(t_{1}<t_{2}|x_{1},x_{2}\right)\\ =\int_{-\infty }^{+\infty }dt_{2}\int_{-\infty }^{t_{2}}dt_{1}\;p\left(t_{1}|x_{1}\right)p\left(t_{2}|x_{2}\right) \end{array}$$

If *p*(*D* = 0|*x*_1_, *x*_2_) > *p*(*D* = 1|*x*_1_, *x*_2_), the participant reports *t*_1_ as being longer, otherwise he/she reports *t*_2_ as being longer. If Equations (17) and (18) are expanded by plugging in Equation (16), we notice that *p*(*x*_1_)*p*(*x*_2_) is shared in both the formula of *p*(*D* = 0|*x*_1_, *x*_2_) and *p*(*D* = 1|*x*_1_, *x*_2_). Therefore, the terms *p*(*x*_1_) and *p*(*x*_2_) can be ignored in making judgment about *D*.

#### Choice probability

While the inference process described above is deterministic, *x*_1_ and *x*_2_, the measurements of duration based on certain neural processes in the visual pathway are stochastic. They can vary from trial to trial even if the physical durations are the same. In our modeling, this variation was the major source of variability in participants' judgments. We did not make specific assumption on how *x*_1_ and *x*_2_ are generated. We only made the simple assumption that their distributions follow Equations (1) and (2). In order to calculate the probability that a participant makes a certain judgment on a trial, we integrated over the space of distribution of *x*_1_ and *x*_2_ where the corresponding judgment should be made according to the above decision rule. In addition, similarly as in Experiment 1 and 2, we included a lapse rate term which describes the probability that a participant fails to pay attention to the stimuli and makes a random guess. The choice probability thus takes the following form:

(19) $$p_{M,\theta }\left(r|t_{1},t_{2}\right)=\{\begin{array}{c} \begin{array}{c} \frac{1}{2}\lambda +\;\left(1-\lambda \right)\int_{-\infty }^{+\infty }dx_{1}\int_{-\infty }^{+\infty }dx_{2}\text{H}\left(p_{M,\theta }\left(D=1|x_{1},x_{2}\right)-p_{M,\theta }\left(D=0|x_{1},x_{2}\right)\right)\\ \cdot \;p_{M,\theta }\left(x_{1}|t_{1}\right)\cdot p_{M,\theta }\left(x_{2}|t_{2}\right),\text{ if }r="{\text{t}}_{2}\text{ is longer}" \end{array}\\ \begin{array}{c} \frac{1}{2}\lambda +\;\left(1-\lambda \right)\int_{-\infty }^{+\infty }dx_{1}\int_{-\infty }^{+\infty }dx_{2}\text{H}\left(p_{M,\theta }\left(D=0|x_{1},x_{2}\right)-p_{M,\theta }\left(D=1|x_{1},x_{2}\right)\right)\\ \cdot \;p_{M,\theta }\left(x_{1}|t_{1}\right)\cdot p_{M,\theta }\left(x_{2}|t_{2}\right),\text{ if }r="t_{2}\text{ is shorter}" \end{array} \end{array}$$

In the above equation, *r* is the judgment. *M* indicates the model under consideration. **θ** represents all the free parameters of model *M*. H(·) means a step function which outputs 1 only when the input is larger or equal to 0 and outputs 0 otherwise. λ is the lapse rate.

An analytic form of the choice probability does not exist as function of *t*_1_ and *t*_2_. To calculate the integral numerically, we used a Gaussian–Hermite quadrature of order 7 to approximate the integration over *x*_1_. For a value of *x*_1_ chosen as the abscissa in the integration, the value of *x*_2_ that satisfies *p*(*D* = 0 |*x*_1_, *x*_2_) = *p*(*D* = 1 |*x*_1_, *x*_2_) was found by numerical search. The step function H(·) is 1 on one side of this value of *x*_2_ and 0 on the other side. Therefore, the integration over *x*_2_ was calculated based on the cumulative distribution function of *p*(*x*_2_ |*t*_2_) at this value of *x*_2_.

#### Model comparison

Our goal was to understand how the brain forms a duration representation when multiple stimuli, each providing conflicting evidence of duration occur simultaneously. In our modeling framework, the process of forming duration representation based on multiple stimuli is the process of calculating the likelihood of a duration *t* when the stimulus is HL. Thus, one major difference between the models under consideration is in their likelihood function *p*(*x*_*i, L*_, *x*_*i, H*_ |*t*_*i*_) (*i* = 1, 2), when the stimulus in *t*_*i*_ is HL and separate sensory measurements *x*_*i, L*_ and *x*_*i, H*_ are formed. In addition, we also aimed to understand the discrepancy observed in the psychometric curves corresponding to different orders of displaying the reference and comparison stimuli. We considered two possible causes for the discrepancy: the sensory measurement of the first duration on a trial may be degraded more than that of the second due to decay of memory, and participants may incorporate the prior belief of duration distribution into their inference process.

Therefore, we constructed models based on three factors: the likelihood function of duration when the stimulus is HL, whether memory decay exists, and how participants incorporate prior belief of stimulus duration during inference.

##### Likelihood function

The form of the likelihood function of duration t when the stimulus is H or L is shared among all models. As the distribution of measurement *x* has a constant level of noise over the range of *t* (on log scale), a reasonable assumption is that the likelihood function follows the shape of Gaussian function with the same standard deviation as the level of noise:

(20) $$L\left(t_{i}\right)=p(x_{i}|t_{i})=\{\begin{array}{c} \text{N}(x_{i},\sigma_{\text{H}}),\text{ if H stimulus is displayed}\\ \text{N}(x_{i},\sigma_{\text{L}}),\text{ if L stimulus is displayed} \end{array}$$

In the above equation, we also assumed that the biases *b*_H_ and *b*_L_ in the distributions of *x*_H_ or *x*_L_, as in Equation (1) and (2), are not accessible by the brain at the inferring stage. This assumption and the difference between *b*_H_ and *b*_L_ explain why H is judged as longer than L in our modeling framework.

The likelihood function of duration *t* when the stimulus is HL differs between models.

In models assuming the “weighting” hypothesis, we assume that the brain first weights the two sensory measurements of duration by Equation (3). The likelihood function of *t* is then calculated based on *x*_HL_:

(21) $$\begin{array}{l} \;p\left(x_{i,\text{L}}x_{i,\text{H}}|t_{i}\right)=L_{\text{weighting}}\left(t_{i}\right)\\ =N(t_{i};w_{\text{H}}x_{i,\text{H}}+(1-w_{\text{H}})x_{i,\text{H}},\sqrt{w_{\text{H}}^{2}\sigma_{i,\text{H}}^{2}+{\left(1-w_{\text{H}}\right)}^{2}\sigma_{i,\text{L}}^{2}}) \end{array}$$

We modeled the standard deviation of the likelihood function as in the above equation because it matches the standard deviation of the distribution of *x*_HL_ following the weighting scheme in Equation (3).

In models assuming the “optimal integration” hypothesis, a stronger version of the “weighting” hypothesis, the likelihood is the product of the likelihood of *t* based on each individual stimulus element, which amounts to:

$$p\left(x_{i,\text{L}}x_{i,\text{H}}|t_{i}\right)=L_{\text{optimal}}\left(t_{i}\right)=N\left(x_{i,\text{H}},\sigma_{\text{H}}\right)\cdot N\left(x_{i,\text{L}},\sigma_{\text{L}}\right)$$

In models assuming the “selection” hypothesis, the likelihood function is based only on the stimulus element that is selected to estimate duration:

(22) $$\begin{array}{l} p\left(x_{i,\text{L}}x_{i,\text{H}}|t_{i}\right)\text{​}=\text{​}L_{\text{selection}}\left(t_{i}\right)\\ \text{ ​}=\text{​}\{\text{​}\begin{array}{c} N\left(x_{i,\text{H}},\sigma_{\text{H}}\right)\text{​},\text{if stimulus H is selected}\\ \text{​}N\left(x_{i,\text{L}},\sigma_{\text{L}}\right)\text{​},\text{if stimulus L is selected} \end{array} \end{array}$$

In models assuming the “reliable stimulus” hypothesis, the likelihood function is based on the stimulus element which the participants has a smaller standard deviation in his/her estimation of duration:

(23) $$\begin{array}{l} p\left(x_{i,\text{L}}x_{i,\text{H}}|t_{i}\right)=L_{\text{reliable stimulus}}\left(t_{i}\right)\\ \;=\{\begin{array}{c} N\left(x_{i,\text{H}},\sigma_{\text{H}}\right),\text{ if }\sigma_{\text{H}}<\sigma_{\text{L}}\\ \text{​}N\left(x_{i,\text{L}},\sigma_{\text{L}}\right),\text{ if }\sigma_{\text{H}}>\sigma_{\text{L}} \end{array} \end{array}$$

In models assuming the “weighting,” “optimal integration,” or “reliable stimulus” hypothesis, the likelihood function can be plugged into the inference process and the choice probability can be calculated for each combination of model parameters.

In models assuming the “selection” hypothesis, if the reference stimulus is HL and the comparison stimulus is H or L, then the two choice probabilities, corresponding to either H or L element being selected from the reference stimulus, are first calculated by plugging the likelihood function corresponding to that stimulus being selected into the inference process. Then these probabilities are further multiplied by the probabilities of H or L being selected and summed together, to calculate the expected choice probability for a given trial.

(24) $$\begin{array}{l} p\left(r|t_{1},t_{2},\theta ,\text{M}\right)=p_{\text{select H}}\left(r|t_{1},t_{2},\theta ,\text{M}\right)c_{\text{H}}\\ \;+\;p_{\text{select L}}\left(r|t_{1},t_{2},\theta ,\text{M}\right)(1-c_{\text{H}}) \end{array}$$

If the comparison stimulus is also HL, then the equation above is used to first calculate the choice probabilities of either H or L element being selected from the comparison stimulus. They are further multiplied by *c*_H_ and 1-*c*_H_ and summed similarly.

##### Memory decay

In order to make a comparison of duration, participants need to hold the memory of the duration of the first stimulus until making judgment. At the time of making judgment, more time had elapsed since the first stimulus than since the second stimulus. It is possible that the representation of duration of the first stimulus was more variable than that of the second stimulus due to decay of memory. Therefore, the second factor that we consider in constructing models is whether the standard deviation of *x*_1_ increases compared to *x*_2_ due to memory decay.

In models assuming the “decay” hypothesis, the standard deviation of the distribution of *x*_1_ is scaled up by a constant *m* (*m* > 1) relative to that of *x*_2_ of the same type of stimulus. *m* is a free parameter common to all stimulus types. The standard deviation of the likelihood function of the first duration *t*_1_ is also scaled up by *m*.

In models assuming the “no decay” hypothesis, there is no difference in the standard deviation of the distributions of *x*_1_ and *x*_2_, which is equivalent to fixing *m* as 1.

##### Incorporation of prior distribution

The distribution of duration presented in the experiment was not uniform. It is possible that the brain can gradually learn the distribution of duration as the experiment continues. Furthermore, as illustrated in Figures 3B,C, the physical durations of the two stimuli in any trial were sampled from two different distributions with random orders. The brain might further learn this structure. Therefore, we considered three different hypotheses of how the brain might form a belief of the prior distribution of duration.

In models assuming the “flat prior” hypothesis, the brain does not learn any distribution from the experiment but instead assumes any duration is equally possible to occur for both the first and second stimuli. This is equivalent to saying that the posterior of duration is the same as the likelihood of duration: *p*(*t*_*i*_ |*x*_*i*_) = *p*(*x*_*i*_ |*t*_*i*_). The generative model assumed by the brain would be without the parameter of displaying order *O* in Figure 3B.

In models assuming the “single prior” hypothesis, the brain forms a belief that all stimulus durations are sampled from the same distribution, which is the mixture of the distribution of the reference and comparison duration. Note that it is impossible for participants to learn the exact distribution of the physical duration, because of the noise in sensory measurement of duration, and because H and L type of stimuli cast different biases on the measurements. Therefore, the prior distribution learnt by the brain should be a smoothed version of the true distribution. For simplicity, we assume that the prior distribution *p*(*t*_*i*_) in Equation (16) takes the form of the convolution of a Gaussian kernel with the mixture of distributions of the true duration of both the reference and comparison stimuli.

In models assuming the “double priors” hypothesis, the brain learns the correct generative model as in Figure 3C, that durations are sampled from two distributions and a top-level variable *O* determines the order in which the two durations are drawn from these distributions. In order to account for both the possible orders of display, the brain separately calculates the posterior probabilities of the decision variable *D* based on each possible order *O*, and marginalize over *O* by taking the average of these two probabilities:

(25) $$p\left(D=0|x_{1},x_{2}\right)=\frac{\begin{array}{l} p\left(t_{1}>t_{2}|x_{1},x_{2},O=\;"\text{c-r}"\right)\\ +p\left(t_{1}>t_{2}|x_{1},x_{2},O=\;"\text{r-c}"\right) \end{array}}{2}$$

(26) $$p\left(D=1|x_{1},x_{2}\right)=\frac{\begin{array}{l} p\left(t_{1}<t_{2}|x_{1},x_{2},O=\;"\text{c-r}"\right)\\ +\;p\left(t_{1}<t_{2}|x_{1},x_{2},O=\;"\text{r-c}"\right) \end{array}}{2}$$

In the above equations, *p*(*t*_1_ > *t*_2_|x_1_, x_2_, *O*) and *p*(*t*_1_ < *t*_2_|x_1_, x_2_, *O*) were calculated similarly as in Equation (17), except that the posterior probabilities of *t*_1_ and *t*_2_ depend on the variable *O*. We named the prior probability of the duration of the comparison stimuli by *p*_*c*_(*t*), and that of the reference stimuli by *p*_*r*_(*t*). The posterior probabilities of *t*_1_ and *t*_2_ corresponding to the two orders of display are:

(27) $$\begin{array}{l} p(t_{1}|x_{1},x_{2},O="\text{c-r}")=\frac{p_{\text{c}}(t_{1})p(x_{1}|t_{1})}{p(x_{1})},\\ p(t_{2}|x_{1},x_{2},O="\text{c-r}")=\frac{p_{\text{r}}(t_{2})p(x_{2}|t_{2})}{p(x_{2})} \end{array}$$

(28) $$\begin{array}{l} p(t_{1}|x_{1},x_{2},O="\text{r-c}")=\frac{p_{\text{r}}(t_{1})p(x_{1}|t_{1})}{p(x_{1})},\\ p(t_{2}|x_{1},x_{2},O="\text{r-c}")=\frac{p_{\text{c}}(t_{2})p(x_{2}|t_{2})}{p(x_{2})} \end{array}$$

As described above, we considered three factors: the mechanism of combining duration estimates based on simultaneous stimuli, the existence of memory decay, and the form of prior distribution. Each combination of these three factors generates one model. We compared 24 models (4 × 2 × 3) in total based on cross-validated log-likelihoods of the models (van den Berg et al., 2014). We first separated the trials of each participant into 12 subsets. Each subsets contained approximately an equal number of trials belonging to each condition and each order of display (we say “approximately” because the total number of trials is not a multiple of 12). Then for each model, we performed 12-fold cross validation. In each case, we left one subset of trials out as testing data. Trials of the other 11 subsets were treated as training data. We fitted the model to the training data by searching for a combination of parameters that maximizes the product of the choice probabilities over all trials in the training data. Then with parameters fitted to the training data, we calculated the log-likelihood of the testing data as the logarithm of the product of the choice probabilities over all trials in the testing data. The sum of the log-likelihoods of the testing data over the 12 instances of cross-validation is the cross-validated log-likelihood of the model being compared. Figure 3D illustrate this procedure.

### Conflict of interest statement

The authors declare that the research was conducted in the absence of any commercial or financial relationships that could be construed as a potential conflict of interest.
